# Ninth International Medical Congress

**Published:** 1885-11

**Authors:** 


### Editorial, Etc.

---

NINTH INTERNATIONAL MEDICAL CONGRESS, WASHINGTON, D. C., 1887.—[The following are some of the more important Rules adopted relating to the preliminary organization of the Congress.—ED.]

1. The Congress shall consist of members of the regular profession of medicine, who shall have inscribed their names on the register and shall have taken out their tickets of admission; and of such other scientific men as the Executive Committee of the Congress may see fit to admit.

2. The dues for members of the Congress shall be ten dollars each for members residing in the United States.

There shall be no dues for members residing in foreign countries.

Each member of the Congress shall be entitled to receive a copy of the "Transactions" for 1887.

3. The Congress shall be divided as follows, into seventeen Sections:

I. General Medicine.
II. General Surgery.
III. Military and Naval Surgery.
IV. Obstetrics.
V. Gynæcology.
VI. Therapeutics and Materia Medica.
VII. Anatomy.
VIII. Physiology.
IX. Pathology.
X. Diseases of Children.
XI. Ophthalmology.
XII. Otology and Laryngology.
XIII. Dermatology and Syphilis.
XIV. Public and International Hygiene.
XV. Collective Investigation Nomenclature, Vital Statistics, and Climatology.
XVI. Psychological Medicine and Diseases of the Nervous System.
XVII. Dental and Oral Surgery.

4. The General Meetings of the Congress shall be for the transaction of business and for addresses and communications of general scientific interest.

8. The official languages of the Congress shall be English, French, and German.

In the meetings of the Sections, no member shall be allowed to speak for more than ten minutes, with the exceptions of the readers of papers and those who introduce subjects for discussion, who may each occupy twenty minutes.

9. The rules and programmes shall be published in English, French and German.

Each paper and address shall be printed in the "Transactions' in the language in which it was presented, and preliminary abstracts of papers and addresses also shall be printed, each in the language in which it is to be delivered

All discussions shall be printed in English.

10. The President of the Congress, the Secretary-General, the Treasurer, the Chairman of the Finance Committee, and the Presidents of the Sections, shall together constitute an an Executive Committee of the Congress, which Committee shall direct the business of the Congress. shall authorize all expenditures for the immediate purposes of the Congress, shall supervise and audit the accounts of the Treasurer, and shall fill all vacancies in the offices of the Congress and of the Sections. This Committee shall have power to add to its membership, but the total number of members shall not exceed thirty. A number equal to one-third of the members of the Committee shall constitute a quorum for the transaction of business.

11. The Officers of the Congress shall be a President, Vice-Presidents, a Secretary-General, four Associate Secretaries, one of whom shall be the French Secretary, and one of whom shall be the German Secretary, a Treasurer, and the Chairman of the Finance Committee.

12. The officers of each Section shall be President, Vice-Presidents, Secretaries, and a Council.

13. The officers of the Congress, and the officers of the Sections, shall be nominated to the Congress at the opening of its first session.

It will be seen by the above that the Section of "Oral and Dental Surgery" has been restored to the position assigned it by the original Committee. A meeting of the Executive Committee will be held in New York, on Wednesday November 18th, 1885.

This Section as originally appointed is as follows:

PRESIDENT.—Jonathan Taft, M. D., D. D. S., Cincinnati, Ohio:

VICE-PRESIDENTS.—W. W. Allport, M. D., D. D. S., Chicago, Ill; William H. Dwinelle, M. D., D. D. S., New York City; J. L. Williams, M. D., D. D. S., Boston, Mass.

SECRETARIES.—E. A. Bogue, M. D., D. D. S., New York City; Geo. H, Cushing, D. D. S., Chicago, Ill.

MEMBERS OF COUNCIL.—W. C. Barrett, M. D., D. D. S., Buffalo, N. Y; Thos. Fillebrown, M. D., D. M. D., Portland, Me; F. J. S. Gorgas, M. D., D. D. S., Baltimore, Md; Edw. Maynard, M. D., Washington, D. C; J. H. McKellops, D. D. S., St. Louis. Mo. W. H. Morgan, M. D., D. D. S., Nashville, Tenn; C. N. Pierce, D. D. S., Philadelphia, Pa; L. D. Shepard, D. D. S., Boston, Mass; J. Truman, D. D. S., Philadelphia, Pa · James W. White, M. D., D. D. S., Philadelphia, Pa.

